# Hypovitaminosis C and vitamin C deficiency in critically ill patients despite recommended enteral and parenteral intakes

**DOI:** 10.1186/s13054-017-1891-y

**Published:** 2017-12-11

**Authors:** Anitra C. Carr, Patrice C. Rosengrave, Simone Bayer, Steve Chambers, Jan Mehrtens, Geoff M. Shaw

**Affiliations:** 1 Department of Pathology, University of Otago, Christchurch, PO Box 4345, Christchurch, 8140 New Zealand; 2 Department of Intensive Care Medicine, Christchurch Hospital, Private Bag 4710, Christchurch, 8140 New Zealand

**Keywords:** Intensive care, Vitamin C, Hypovitaminosis C, Sepsis, Critical illness, C-reactive protein, Enteral nutrition, Parenteral nutrition, Septic shock

## Abstract

**Background:**

Vitamin C is an essential water-soluble nutrient which cannot be synthesised or stored by humans. It is a potent antioxidant with anti-inflammatory and immune-supportive roles. Previous research has indicated that vitamin C levels are depleted in critically ill patients. In this study we have assessed plasma vitamin C concentrations in critically ill patients relative to infection status (septic shock or non-septic) and level of inflammation (C-reactive protein concentrations). Vitamin C status was also assessed relative to daily enteral and parenteral intakes to determine if standard intensive care unit (ICU) nutritional support is adequate to meet the vitamin C needs of critically ill patients.

**Methods:**

Forty-four critically ill patients (24 with septic shock, 17 non-septic, 3 uncategorised) were recruited from the Christchurch Hospital Intensive Care Unit. We measured concentrations of plasma vitamin C and a pro-inflammatory biomarker (C-reactive protein) daily over 4 days and calculated patients’ daily vitamin C intake from the enteral or total parenteral nutrition they received. We compared plasma vitamin C and C-reactive protein concentrations between septic shock and non-septic patients over 4 days using a mixed effects statistical model, and we compared the vitamin C status of the critically ill patients with known vitamin C bioavailability data using a four-parameter log-logistic response model.

**Results:**

Overall, the critically ill patients exhibited hypovitaminosis C (i.e., < 23 μmol/L), with a mean plasma vitamin C concentration of 17.8 ± 8.7 μmol/L; of these, one-third had vitamin C deficiency (i.e., < 11 μmol/L). Patients with hypovitaminosis C had elevated inflammation (C-reactive protein levels; *P* < 0.05). The patients with septic shock had lower vitamin C concentrations and higher C-reactive protein concentrations than the non-septic patients (*P* < 0.05). Nearly 40% of the septic shock patients were deficient in vitamin C, compared with 25% of the non-septic patients. These low vitamin C levels were apparent despite receiving recommended intakes via enteral and/or parenteral nutritional therapy (mean 125 mg/d).

**Conclusions:**

Critically ill patients have low vitamin C concentrations despite receiving standard ICU nutrition. Septic shock patients have significantly depleted vitamin C levels compared with non-septic patients, likely resulting from increased metabolism due to the enhanced inflammatory response observed in septic shock.

## Background

Vitamin C is an essential water-soluble nutrient which cannot be synthesised by humans, owing to loss of the terminal enzyme in the biosynthetic pathway [1]. The vitamin has numerous physiological roles in the body through acting as an electron donor. It is a potent antioxidant, protecting important biomolecules (proteins, lipids and DNA) from oxidation, thus preserving essential tissue structure and function [2]. Vitamin C also acts as an enzyme cofactor for a number of biosynthetic enzymes involved in hormone synthesis and generation of metabolic energy [3], as well as an ever-expanding family of gene-regulatory enzymes [4, 5]. Recent research has indicated important roles for vitamin C in the regulation of gene transcription via transcription factors and epigenetic modifying enzymes [4, 5]. Its function as a cofactor for the metalloenzymes which synthesise the vasopressors noradrenaline and vasopressin suggests an important role in critically ill patients, particularly those with sepsis and septic shock [6]. Several early studies have indicated that critically ill patients, including those with sepsis, exhibit very low circulating vitamin C levels compared with healthy control individuals [7, 8], and deficient levels correlated with multiple organ failure [9]. Owing to the pleiotropic functions of vitamin C in the body, depleted vitamin C status in critically ill patients may compromise their recovery.

Patients in the intensive care unit (ICU) routinely receive enteral nutrition and/or parenteral nutrition for those who cannot receive enteral nutrition or cannot receive it in sufficient amounts [10]. The route of feeding—enteral or parenteral—is determined by the presence or absence of a functioning intestine and the haemodynamic status of the patient [11]. Nutritional requirements are often tailored in the setting of fluid restriction and organ failure [11]. Nutrition therapy provides essential macronutrients and micronutrients, such as vitamins A, C and E, which are thought to help prevent oxidative stress and modulate immune responses [12]. Although total parenteral nutrition (TPN) typically comprises 100 mg/day vitamin C, one study has indicated that restoration of normal circulating vitamin C levels in critically ill patients requires a 30-fold higher intake [13]. This is likely due to the enhanced metabolic demands for vitamin C in these patients owing to inflammatory and/or infectious processes.

However, whether critically ill patients are receiving adequate vitamin C intakes via standard ICU nutritional therapy to meet their enhanced needs remains largely underexplored. We therefore assessed the plasma vitamin C status of critically ill patients in intensive care and related this to infectious status (septic shock or non-septic), inflammation (C-reactive protein concentrations) and daily enteral and/or parenteral intakes.

## Methods

We carried out an observational study with 44 critically ill patients recruited in the ICU of Christchurch Hospital, Christchurch, New Zealand (December 2015–August 2016). All procedures involving human participants were approved by the Southern Health and Disability Ethics Committee (15/STH/36).

All patients aged > 18 years admitted to the ICU were eligible, except those not expected to survive 72 h or when consent could not be obtained. Proxy consent was obtained from the treating physician in consultation with family members when patient consent was not possible. Consent was sought from patients as soon as they had sufficiently recovered. Only one patient withdrew consent; that patient’s data was discarded. The final cohort comprised 44 patients: a septic shock group (*n* = 24), a non-septic group (*n* = 17) and three patients who could not be categorised on the basis of the clinical notes and therefore were not included in subgroup analysis. Inclusion in the septic shock group required meeting the following criteria: receiving ≥ 5 μg/minute noradrenaline or adrenaline, receiving intravenous antimicrobial therapy specifically for infection, evidence of organ dysfunction (i.e., Sequential Organ Failure Assessment [SOFA] score ≥ 2 for at least one of respiratory function [ratio of partial pressure of arterial oxygen and fraction of inspired oxygen < 300], liver function [bilirubin > 33 μmol/L], coagulation [platelets < 100 × 10^3^/μL] and renal function [creatinine > 171 μmol/L or creatinine increase > 50% of baseline if chronic renal failure]). One patient entered the study with unusually high plasma vitamin C concentrations (>100 μmol/L) and was classified as an outlier. This patient was excluded from all analyses involving vitamin C and has been presented separately as a case study.

Several ICU scoring systems were used at enrolment: the Simplified Acute Physiology Score II (SAPS II) [14] and the Acute Physiology and Chronic Health Evaluation (APACHE) II and III scores [15]. Organ failure was assessed using the SOFA score [16] daily for the duration of the study.

### Vitamin C and C-reactive protein analysis

A blood sample (4-ml ethylenediaminetetraacetic acid tube) was collected from each patient through an already established arterial line at enrolment (baseline = 0 h) and at 12, 24, 48, 72 and 96 h. The blood was immediately placed on ice and processed for vitamin C analysis within 2 h of collection by centrifugation at 4 °C to separate plasma, and precipitation of proteins and stabilisation of vitamin C was carried out with perchloric acid and the metal chelator diethylenetriaminepentaacetic acid as described previously [17]. The acidified supernatants were stored at −80 °C for batch analysis. Urine samples were collected from a subset of patients through an indwelling urinary catheter at times 0, 12, 24, 48, 72 and 96 h. Plasma and urine vitamin C concentrations were determined using reversed-phase high-performance liquid chromatography with electrochemical detection as described previously [17]. Half of each acidified sample was treated with the reducing agent tris(2-carboxyethyl)phosphine (TCEP) to determine the amount of dehydroascorbic acid present [18]. Treatment of the samples with the reducing agent TCEP showed no significant gain in vitamin C concentrations, other than two of the samples which exhibited haemolysis. Plasma C-reactive protein concentration was assessed by endpoint nephelometry, and urinary creatinine concentrations were determined by the classical Jaffe reaction at Canterbury Health Laboratories, an International Accreditation New Zealand laboratory.

### Patient vitamin C intake assessment

The daily vitamin C intake of the patients was determined from the volume of enteral nutrition or TPN administered to the patients. The enteral nutrition (Jevity, Glucerna, and Osmolite [Abbott Nutrition, Lake Bluff, IL, USA], Fresubin® 1000 complete and Fresubin® Energy Fibre [Fresenius Kabi, Bad Homburg, Germany]) contained 100–110 mg vitamin C/L, and the TPN (Olimel® N9; Baxter, Old Toongabbie, Australia) contained 62.5 mg vitamin C/L plus an additional 150 mg/L sodium ascorbate, which was included as a pH stabiliser (Baxter Healthcare, Christchurch, NZ), providing a total of 196 mg/L ascorbic acid (vitamin C).

### Statistical analysis

Data are represented as mean and SD or box plots showing median values, 25th and 75th percentiles as boundaries, and whiskers as the range. To compare plasma and urinary vitamin C, as well as C-reactive protein concentrations, in septic shock and non-septic patients over the study period, we performed linear mixed effects models (LMEMs) using the lme4 [19] and lmerTest [20] packages in R version 3.2.4 [21]. Patient identity was included in all models as a random variable to account for repeated measures of the same patient over the 4 days. The Satterthwaite method was used to calculate *P* values from *t* tests. We included an interaction effect in both models of day sampled × patient group (septic shock or non-septic). Post hoc analysis was conducted for each model using the multcomp package in R [22]. Model assumptions were verified. All effects were statistically significant at α = 0.05.

Using the pharmacokinetic data from the study by Levine et al. [23], whereby the steady-state plasma vitamin C concentrations for vitamin C-deficient individuals were determined following vitamin C of doses of 30–1000 mg/day, we constructed a dose-response model. Using this model we estimated plasma vitamin C concentrations for the critically ill patients in this study on the basis of the known daily enteral and/or parenteral nutritional intake they received. To do this we constructed a four-parameter log-logistic response model using the drc package in R [24] and the following function: ((f(x) = c + \frac{d-c}{1 + \exp(b(\log(x)-\log(e)))}). All model assumptions were met.

## Results

### Patient characteristics

During the study period, 44 patients (24 septic shock patients, 17 non-septic patients and 3 uncategorised) were recruited from the ICU. Demographic and clinical characteristics of the recruited patients are shown in Table 1. Of the participants, 61% were male, including 71% and 58% in the non-septic and septic shock groups, respectively. There were no statistically significant differences detected between the septic shock and non-septic patients in terms of demographic data and clinical characteristics. The non-septic group comprised primarily cardiac cases, whereas the sources of infection in the septic shock group were primarily abdominal and respiratory in origin. Severity of illness based on the SOFA, SAPS II and APACHE II scores were comparable between the groups at the time of enrolment.

**Table 1:** Participant characteristics

Characteristic	Total cohort (*n* = 44)*	Non-septic (*n* = 17)	Septic shock (*n* = 24)	*P* value^a^
Gender, % male	61	71	58	
Age, years	64 (14)	64 (11)	64 (15)	0.3
Weight, kg	78 (20)	87 (18)	72 (19)	0.9
Baseline disease				
Cardiac		12		
Neurological		3		
Trauma		1		
Failed renal transplant		1		
Source of sepsis				
Abdominal			13	
Respiratory			8	
Skin/joint			2	
Urinary			1	
SAPS II	45.8 (14)	46.5 (13)	45.3 (16)	0.8
APACHE II	20 (6.4)	21.4 (7.0)	19 (5.9)	0.3
SOFA	8.2 (3.6)	9.4 (3.5)	7.6 (3.2)	0.8

*Abbreviations: SAPS* Simplified Acute Physiology Score, *APACHE* Acute Physiology and Chronic Health Evaluation, *SOFA* Sequential Organ Failure Assessment

Data are presented as percentage or mean and SD where applicable

^*^Note that three patients could not be categorised as either non-septic or septic shock

^a^Unpaired Student’s *t* test for non-septic versus septic shock

### Plasma vitamin C status of critically ill patients

**Fig. 1:**
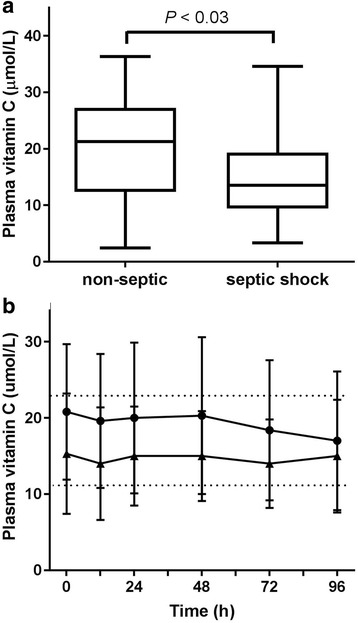
Plasma vitamin C concentrations in critically ill patients. **a** Septic shock patients had significantly lower vitamin C concentrations than non-septic patients (*P* < 0.03 using linear mixed effects model). Box plots show median with 25th and 75th percentiles as boundaries, and whiskers are the range. **b** Time course of plasma vitamin C concentrations in the non-septic group *(filled circles)* and the septic shock group (*filled triangles)*. Data represent mean and SD. *Dotted lines* indicate the hypovitaminosis C cutoff (23 μmol/L) and vitamin C deficiency cutoff (11 μmol/L)

The baseline vitamin C status of the total cohort was 17.8 ± 8.7 μmol/L; of these, 68% were classified as hypovitaminosis C (i.e., < 23 μmol/L), and 32% were deficient in vitamin C (i.e., < 11 μmol/L). Overall, mean plasma vitamin C concentrations were significantly lower in septic shock patients (15.3 ± 7.9 μmol/L) than in the non-septic patients (20.8 ± 8.9 μmol/L; LMEM, *F*_1,38.5_ = 5.1; *P* = 0.03) (Fig. 1a). Of the septic shock patients, 88% were in the hypovitaminosis C category, compared with 50% in the non-septic patients, and 38% of the septic shock patients were deficient in vitamin C, compared with 25% of the non-septic patients. There was no interaction effect on plasma vitamin C levels between day sampled during the study and patient group (septic shock and non-septic; LMEM, F_4,114.4_ = 1.1; *P* = 0.4) (Fig. 1b), suggesting that vitamin C levels did not vary over the days sampled and between patient groups.

### C-reactive protein concentrations relative to plasma vitamin C

**Fig. 2:**
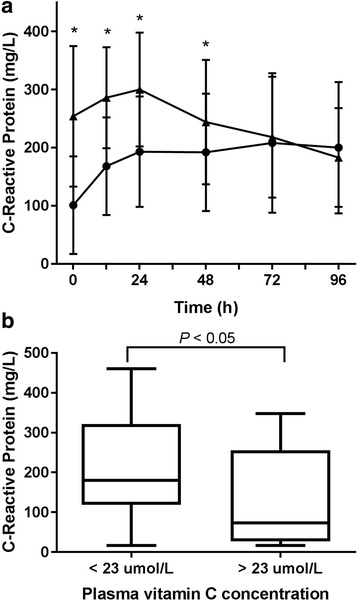
Plasma C-reactive protein concentrations in critically ill patients. **a** Time course of C-reactive protein concentrations in the non-septic group (*filled circles)* and the septic shock group (*filled triangles)*. Data represent mean and SD. Significant differences between the two groups are indicated (*P < 0.01 using linear mixed effects model). **b** C-reactive protein concentrations relative to vitamin C concentrations. Baseline (time 0 h) C-reactive protein concentrations were divided around the hypovitaminosis C value of 23 μmol/L. Box plots show medians with 25th and 75th percentiles as boundaries and whiskers as the range. The C-reactive protein concentration was significantly different between the hypovitaminosis C group and > 23 μmol/L group (*P* < 0.05)

C-reactive protein levels were dependent on the interaction effect between day sampled and patient group (LMEM, *F*_4,115_ = 5.3, *P* = 0.0006). At baseline (time = 0 h), patients with septic shock had 2.4-fold higher C-reactive protein concentrations (254 ± 121 mg/L) than non-septic patients (105 ± 84 mg/L; LMEM, z = 4.4, *P* < 0.001) and remained higher than non-septic patients for the first 48 h (LMEM, z = 5.6–3.9, *P* < 0.001–0.04) (Fig. 2a). After this time, C-reactive protein levels were the same as those of non-septic patients. For non-septic patients, C-reactive protein levels were the lowest at baseline compared with the other time points sampled (LMEM, z = 3.1–3.6*, P* = 0.01–0.04). When baseline C-reactive protein levels were divided around the hypovitaminosis C cutoff, there was a significant difference between the two groups (*P* = 0.05), with the mean C-reactive protein value for the hypovitaminosis C group being 216 ± 122 mg/L and that of the >23 μmol/L group being 130 ± 122 mg/L (Fig. 2b).

### Plasma vitamin C concentrations relative to intakes

Over 80% of the critically ill patients received enteral and/or parenteral nutrition over the duration of the 4-day study period. The average daily intake of vitamin C for those who received enteral and/or parenteral nutrition was 125 ± 88 mg/day (with the maximum intake being 448 mg/day). Of the total cohort, 64% received enteral nutrition only (mean vitamin C intake 102 ± 54 mg/day), 7% received total parenteral nutrition only (mean vitamin C intake 206 ± 106 mg/day) and 10% received a combination of enteral and parenteral (mean vitamin C intake 195 ± 144 mg/day).

**Fig. 3:**
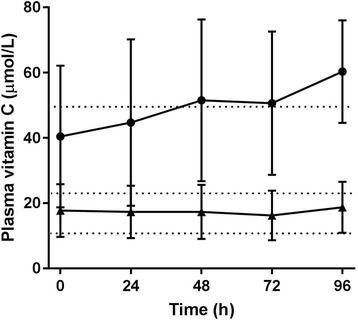
Predicted compared with measured vitamin C concentrations in critically ill patients. Vitamin C concentrations predicted from enteral and/or parenteral administration (*filled circles*) were compared with measured plasma vitamin C concentrations (*filled triangles*). Predicted levels were obtained by fitting a four-parameter log-logistic response model to the pharmacokinetic data reported elsewhere [23]. Data represent mean and SD. All measured values were significantly lower than predicted values (*P* < 0.0001). *Dotted lines* indicate inadequate vitamin C cutoff (50 μmol/L), hypovitaminosis C cutoff (23 μmol/L) and vitamin C deficiency cutoff (11 μmol/L)

Using the pharmacokinetic data from the study by Levine et al. [23], we constructed a four-parameter log-logistic response model to predict the plasma vitamin C concentrations for the critically ill patients on the basis of their enteral and/or parenteral vitamin C intakes. The predicted plasma concentrations, although variable, were significantly higher at all time points than the measured plasma concentrations (*P* < 0.0001; Fig. 3). On average, the measured plasma vitamin C values were approximately one-third of the values predicted from intake.

There are a number of possible explanations for the discrepancy in predicted and measured vitamin C concentrations, such as decreased absorption due to gastrointestinal dysfunction in the critically ill patients. However, the patients who were receiving TPN also exhibited hypovitaminosis C (i.e., 16.6 ± 7.5 μmol/L). Another explanation for the difference between predicted and measured vitamin C concentrations is haemodilution due to increased volume of distribution of vitamin C because of the relatively higher water content in critically ill patients after fluid resuscitation. When haemodilution was estimated by comparing the patients’ haematocrit with gender-specific norms, this accounted for 25–34% of the difference (data not shown). Dialysis can also decrease plasma vitamin C levels by up to one-half; however, only five patients were on dialysis during the study period (two non-septic and three septic shock patients), thus, cannot account for the low vitamin C concentrations in the other patients.

### Urinary excretion of vitamin C

Enhanced urinary excretion of vitamin C due to glomerular hyperfiltration and/or dysfunctional tubular reabsorption could potentially result in depleted plasma levels. Therefore, we assessed urinary excretion of vitamin C in a subset of the participants (*n* = 11, comprising 6 male and 5 female, 7 septic shock and 3 non-septic). Urine samples were collected daily at the time of blood sampling. Baseline urinary vitamin C concentrations were 41 ± 30 μmol/L, which increased by up to twofold, depending on the time sampled (LMEM, *F*_1,39_ = 2.5, *P* = 0.05). These concentrations are lower than we have observed previously in healthy participants (baseline 154 μmol/L [25]). Urinary vitamin C concentrations were standardised to urinary creatinine, which decreased by up to half over the 4 days, from a baseline value of 10.4 ± 2.9 mmol/L (LMEM, *F*_1,38_ = 6.1, *P* < 0.001). Thus, urinary vitamin C excretion increased twofold over time relative to urinary creatinine, from a baseline ratio of 4.2 ± 2.9 (LMEM, *F*_1,39_ = 2.5, *P* = 0.005). Urinary output doubled over the study period from a baseline value of 1114 ± 591 ml/day (LMEM, *F*_4,31_ = 2.7, *P* = 0.05). Although 24-h urine collections were not undertaken for vitamin C analysis, we estimated the total vitamin C excreted on the basis of urinary output and related this to the amount of vitamin C administered to the 11 patients in their enteral and parenteral nutrition. Over the study period the patients were administered, on average, 80–122 mg/day vitamin C and excreted, on average, 8–31 mg/day vitamin C. Overall, the patients excreted approximately 15–30% of their administered vitamin C dose over the duration of the study.

### Rapid vitamin C loss in a critically ill patient

**Fig. 4:**
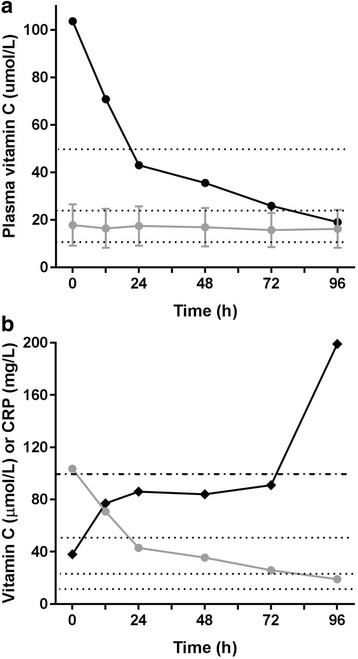
Rapid loss of vitamin C in a critically ill patient. **a** The patient’s vitamin C concentrations (*black circles*) were compared with the critically ill cohort (*grey circles*) over the 4-day study period. Data for the critically ill cohort represents mean and SD (*n* = 43). **b** Increasing C-reactive protein (CRP) concentrations (*filled diamonds*) relative to decreasing vitamin C concentrations (*grey circles*). *Dotted lines* indicate inadequate vitamin C cutoff (50 μmol/L), hypovitaminosis C cutoff (23 μmol/L) and vitamin C deficiency cutoff (11 μmol/L); *dashed line* indicates infectious disease cutoff for CRP (100 mg/L)

One patient entered the study with a very high plasma vitamin C concentration of 104 μmol/L. The patient had been admitted to the ICU following a failed kidney transplant operation. On admission to the ICU the patient was commenced on continuous venovenous haemodiafiltration for acute renal failure. As shown in Fig. 4a, the patient’s initially high vitamin C concentrations dropped to 44 μmol/L within 24 h and continued to decrease to hypovitaminosis C values over the next 3 days in the ICU. Figure 4b shows increasing C-reactive protein concentrations with decreasing vitamin C. On day 4 plasma vitamin C had dropped into the hypovitaminosis C range, and C-reactive protein levels had spiked above 100 mg/L, which is consistent with infection. One week later, the patient became febrile and was diagnosed with ventilator-associated pneumonia (*Staphylococcus aureus*), multiple septic emboli in the lungs, and urinary infection (*Escherichia coli*), and the patient had wound dehiscence. The patient’s condition deteriorated, with fast atrial fibrillation and increasing vasopressor requirements (noradrenaline and vasopressin), and the patient died 2 days later.

## Discussion

Our study revealed that nearly 70% of critically ill patients had hypovitaminosis C, including a high percentage with vitamin C deficiency (32%), despite receiving standard ICU nutritional support. Patients with septic shock have further depletion of plasma vitamin C, with nearly 90% having hypovitaminosis C and 40% having vitamin C deficiency, likely owing to enhanced activation of inflammatory pathways in response to infection, as reflected by higher C-reactive protein levels in these patients. This level of deficiency is significantly greater than we have observed in a middle-aged community-dwelling cohort (*n* = 369), which exhibited < 13% hypovitaminosis C and only 2.4% deficiency [26]. On average, the plasma vitamin C values of those receiving enteral and parenteral nutrition were approximately one-third the values predicted from intake, suggesting that recommended ICU intakes are not sufficient to meet the needs of critically ill patients. It should be noted that the pharmacokinetic data used to calculate the predicted vitamin C concentrations in our participants provide a conservative estimate, because all of the individuals in the pharmacokinetic study had vitamin C deficiency prior to supplementation [23], so the difference between measured and predicted vitamin C concentrations in our participants may be even greater.

There are a number of possible explanations for the discrepancy in predicted and measured vitamin C concentrations. It is possible that enteral and parenteral preparations contain less vitamin C than stated, owing to the instability of the vitamin in the presence of oxygen, transition metal ions, ultraviolet light and higher temperatures [27, 28]. Furthermore, critically ill patients are at significant risk for gastrointestinal dysfunction and consequently poor absorption for those patients who receive enteral feeding [29]. Critically ill patients often exhibit gastrointestinal dysmotility, as well as feeding intolerance such as vomiting, aspiration of gastric contents, or high gastric residual volumes. Patients who are receiving sedatives and vasopressors are also at risk of feeding intolerance, and gastroparesis can be caused by pharmacologic agents that are frequently used in the ICU, such as opioids [30]. Additionally, gastric hypoperfusion in the context of shock or vasopressor use will contribute to poor nutritional uptake of essential vitamins [31]. Although early parenteral nutrition is discouraged in critically ill patients owing to increased risk of infection [31], parenteral feeding bypasses any issues with gastrointestinal dysfunction. Parenteral nutrition also bypasses the saturable intestinal uptake of orally administered vitamin C via sodium-dependent vitamin C transporter 1 (SVCT1) [23]. However, even the participants in our study receiving TPN, which provided an average of approximately 200 mg/day vitamin C, exhibited hypovitaminosis C.

Fluid resuscitation may haemodilute the plasma concentration of vitamin C. When we estimated haemodilution by comparing the patients’ haematocrit with gender-specific norms, this accounted for only one-fourth to one-third of the difference between the measured and predicted vitamin C status. However, this correction is likely an overestimate because critically ill patients also have lower haemoglobin levels owing to inflammation-induced suppression of synthesis and blood sampling or loss. Vitamin C removal from the plasma via dialysis can also occur, and at least 50% may be lost during continuous renal replacement therapy [32–34]. Our study cohort comprised only five patients who were on continuous venovenous haemodiafiltration for acute renal dysfunction whilst in the ICU. One of these patients was presented as a case study, highlighting a rapid and > 50% drop in vitamin C over the first 24 h in the ICU. Depleted plasma vitamin C status might also be due to glomerular hyperfiltration and/or dysfunctional tubular reabsorption of the vitamin via SVCT1 [35]. However, assessment of a subset of patients indicated a lower urinary concentration of vitamin C than we have previously observed in healthy individuals [25]. Furthermore, estimation of total vitamin C excretion based on urinary output provided values that were comparable to those of healthy individuals, who excrete 25% and 50% of a 100-mg dose (for males and females, respectively) [23, 36]. Thus, these estimates suggest that our subset of critically ill patients with low plasma vitamin C concentrations were not excreting more vitamin C than healthy individuals.

Vitamin C acts as a potent antioxidant in vivo, scavenging a wide range of reactive oxygen and nitrogen species, and in the process becoming oxidised to dehydroascorbic acid [2]. The levels of dehydroascorbic acid detected in plasma are normally very low [7, 37]. Although some studies have shown higher levels of dehydroascorbic acid in samples collected from patients assumed to be under enhanced oxidative stress [38–40], we did not observe a significant increase in total vitamin C when our samples were treated with a reducing agent. This indicates that dehydroascorbic acid is not present in the circulation in significant amounts, even in critically ill patients when blood samples are collected and processed in a manner which minimises ex vivo oxidation of vitamin C.

Several studies have shown subnormal vitamin C status in critically ill patients, and this correlated with the severity of the illness and with multiple organ failure [7, 9, 13]. It is likely that the low plasma vitamin C levels in these patients are a marker for correspondingly low tissue status, because we and others have shown that tissue vitamin C status correlates closely with plasma vitamin C levels, both in humans and in animal models [41, 42]. In the present study, we were able to compare patients with septic shock and non-septic patients and found that a greater proportion of patients with septic shock had hypovitaminosis C and vitamin C deficiency. This is likely due to enhanced metabolic turnover as a result of the elevated inflammatory response observed in septic shock. Indeed, the patients with septic shock in our cohort had higher C-reactive protein levels than the non-septic patients for the first 48 h of the study. C-reactive protein is an acute-phase protein synthesised by the liver during acute inflammation in response to infection, trauma, ischaemia and burns along with other inflammatory conditions, and it is often used as a biomarker of sepsis with high levels linked to higher incidences of organ failure and mortality [43]. In our study, significantly higher C-reactive protein levels were observed in patients with lower vitamin C status, which has been observed previously in hospitalised patients [44]. Furthermore, administration of vitamin C to septic patients has been shown to lower C-reactive protein and other pro-inflammatory biomarkers, as well as to decrease organ failure [45, 46].

The American Medical Association’s Nutrition Advisory Board recommends an intake of 100 mg/day vitamin C via liquid nutrition [47]. However, hypovitaminosis C was not prevented in our patients with the use of standard enteral and/or parenteral nutrition, despite our TPN containing additional vitamin C (i.e., a total of nearly 200 mg/L). These results agree with data reported by others who have shown that parenteral intakes of ~ 300 mg/day did not change their patients’ plasma vitamin C concentrations [13, 27]. In comparison, intakes of 150–200 mg/day will saturate the plasma of healthy individuals [23], although we and others have observed decreased response to supplementation in individuals with hypovitaminosis C [48, 49]. Uncomplicated surgical patients require > 500 mg/day of vitamin C, with much higher doses required in surgical ICU patients [50]. Parentaeral administration of ≥ 1000 mg/day vitamin C to critically ill patients was barely sufficient to raise plasma vitamin C concentrations above hypovitaminosis C levels [13, 27]. Recent pharmacokinetic data in critically ill patients indicate that ≥ 2000 mg/day is required to normalise plasma vitamin C levels [51], and 3000 mg/day has been shown to result in saturating levels (i.e., 68 μmol/L), as evidenced by enhanced urinary excretion [13].

Vitamin C can be metabolised via dehydroascorbic acid to oxalate, which has the potential to form calcium oxalate crystals in individuals predisposed to renal stone formation. A few cases of acute oxalate nephropathy have been reported over the last 30 years in patients with impaired renal function following administration of high-gram doses of intravenous vitamin C [52–54]. Administration of gram doses of vitamin C to individuals with normal renal function, however, results in < 0.2% oxalate excretion when samples are collected and stored in a manner which avoids artefactual oxidation [55]. Furthermore, no cases of oxalate nephropathy have been reported for low-gram doses of parenteral vitamin C.

## Conclusions

We have shown that critically ill patients have very low plasma levels of vitamin C, with septic shock patients being particularly depleted, likely resulting from increased metabolism due to the enhanced inflammatory response observed in septic shock. Standard enteral and parenteral nutrition providing an average of 100 and 200 mg/day vitamin C, respectively, is insufficient to meet the needs of critically ill patients. It is likely that vitamin C doses of at least 2–3 g/day are required for these patients. It is also important to note that administration of intravenous rather than oral vitamin C may be required for optimal vitamin C status in these patients because this route bypasses the saturable intestinal uptake of orally administered vitamin C via SVCT1. Further studies assessing the optimal intake and the route of administration of vitamin C for critically ill patients in relation to physiological changes and clinically relevant outcomes are warranted.

## Abbreviations

APACHE: Acute Physiology and Chronic Health Evaluation
CRP: C-reactive protein
ICU: Intensive care unit
LMEM: Linear mixed effects model
SAPS: Simplified Acute Physiology Score
SOFA: Sequential Organ Failure Assessment
SVCT: Sodium-dependent vitamin C transporter
TCEP: Tris(2-carboxyethyl)phosphine
TPN: Total parenteral nutrition
